# Healthcare utilization of breast cancer patients following telephone-based consultations of oncology nurse navigator via telemedical care

**DOI:** 10.1371/journal.pone.0216365

**Published:** 2019-05-02

**Authors:** Gila Adler, Galit Kaufman, Tzahit Simon-Tuval

**Affiliations:** 1 Department of Health Systems Management, Guilford Glazer Faculty of Business and Management and Faculty of Health Sciences, Ben-Gurion University of the Negev, Beer-Sheva, Israel; 2 Maccabi Healthcare Services, Jerusalem and Shfela District, Modi’in-Maccabim-Re’ut, Israel; 3 Maccabi Healthcare Services, Northern District, Haifa, Israel; University of Oxford, UNITED KINGDOM

## Abstract

**Objectives:**

To characterize breast cancer patients who received telephone-based consultations of oncology nurse navigator via telemedical care (TMC patients) and analyze their healthcare utilization (HCU) one year before and after receiving this service.

**Methods:**

A retrospective study among Maccabi Healthcare Services enrollees that were newly diagnosed during 2016 (n = 1035). HCU, demographic characteristics and comorbidities were obtained from computerized database. Multivariable ordered logit model was specified for the determinants of HCU by quarters. Independent variables included: annual number of telephone-based consultations, gap between diagnosis and first consultation, age, socio-economic status, eligibility for disability and income security benefits, and comorbidities.

**Results:**

Twenty-two percent of our cohort were TMC patients. Compared to others, these patients were younger and had a lower prevalence of hypertension. A higher proportion of these patients received disability benefits, and a lower proportion received income security benefits. The total average annual HCU of TMC patients (n = 107) before first consultation was $8857 and increased to $44130 in the first year following it (p<0.001), predominantly due to a significant increase in outpatient visits ($20380 vs. $3502, p<0.001) and medication costs ($19339 vs. $1758, p<0.001). The multivariable model revealed that each additional telephone-based consultation decreased the likelihood to be in the lowest quarter of the HCU distribution by 1.1 percentage points (p = 0.015), and increased the likelihood to be in the upper quarter of the HCU distribution by 1.1 percentage points (p = 0.016).

**Conclusions:**

There was a significant increase in outpatient care and medications usage following first consultation. Moreover, a more intense use of this service was associated with elevated HCU. This result may stem from the proactive nature of the telemedical care.

## Introduction

Cancer, as the leading cause of morbidity and mortality in developed countries [1] imposes a heavy burden on health systems and economies [2–11]. There is evidence that the first year after diagnosis is the costliest year in terms of healthcare utilization (HCU) [9–11]. Outpatient care is a substantial component of HCU [3, 7], and as expected, costs increase as the disease progresses [9, 10]. Diagnosis at early stages of breast cancer leads to better outcomes in terms of higher survival rates and lower costs of care [10, 11]. Potential cost savings associated with early diagnosis is greater in younger patients ages 18–64 than in those of older ages ≥65 [11]. Thus, consideration of primary prevention methods, allocation of coordinated economic resources, and planning for disease management for both secondary and tertiary prevention are essential.

Oncology nurse navigators (ONNs) act as coordinators in the healthcare system by supporting and accompanying cancer patient through their continuity of care during an illness. They provide emotional relief, coordinate services to support social needs, and answer questions; thus, they improve patient satisfaction, health outcomes, and decrease costs both to patients and payers [12]. Improving coordinating mechanisms for hospital–community oncologic care is warranted to improve disease management. These mechanisms may rely on patients and families who will be able to become their own case managers, patients and providers who will informally communicate, and specialist nurses who will be more central in this collaborative process [13]. The current study will focus on the third component—the role of the nurse specialist.

In areas far from healthcare centers, cancer care is associated with unique barriers, including: financial burden of travel and lodging, disruption to family life, lack of care options, difficulty accessing information, and unmet psychological needs. It was found that the timing of the relationship, the ease of accessibility, the information provided, and support offered were valuable in facilitating patients’ treatment and recovery [14–17]. The greatest value of an ONN in the community was found during early cancer detection while focusing on breast cancer prevention through improved mammography adherence [18, 19].

However, the influence of the ONN on patients’ HCU is not consistent. On the one hand, there is evidence that exposure to a professional ONN was associated with a decrease in hospitalization of head and neck cancer patients [20]; on the other hand, no significant differences were observed in charges and reimbursements for hospitalizations, outpatient visits, home care, or emergency room visits of woman diagnosed with breast cancer [21].

Nowadays, technological support such as telehealth services can effectively overcome barriers of distance, time, and cost. Through this technology, patients receive a quick and advanced service from professional health staff. According to a systematic review of reviews, 26% of the reviews concluded that telemedicine is effective (reduced hospitalizations, and improved patient compliance, satisfaction, and quality of life), 23% found that evidence is promising but incomplete, and others that evidence is inconsistent and limited [22]. When comparing live group sessions to an audiovisual technology-based session for breast cancer patients, no significant differences were found between the groups in terms of quality of life, fatigue, cognitive function, spirituality, distress, and sleep. Thus, again, it was concluded that it seems that telehealth-based interventions may represent an effective and feasible strategy for cancer survivors in remote areas [23]. Another systematic review that was focused on different follow-up technologies for patients with cancer (mostly among patients with breast cancer and with regard to telephone follow-up) suggested that these interventions had the same patient satisfaction and safety levels as measured by symptoms, health-related quality of life, and psychological distress as face-to-face visits [24]. However, there is insufficient evidence to evaluate the cost-effectiveness of this care option [24].

In 2012, Maccabi Healthcare Services (MHS) founded their Telemedical Care (TMC) center, a multi-disciplinary healthcare service providing care to complex chronic patients. ONN-led telephone follow-up is provided through this platform as well. Recent research in MHS revealed that TMC services for elderly patients that provide proactive empowerment for self-management can improve the quality of care and is also cost-effective [25]. TMC is especially suitable for frail, home-bound patients with complicated medical conditions [25, 26], and for patients in rural areas where fewer health centers are available [22, 25]. Modern technologies can be used to safely and effectively follow up as an alternative to traditional models that require patients to travel to tertiary cancer centers for face-to-face visits [24]. An economic evaluation found that compared to hospital-based consultations, nurse-led telephone follow-up resulted in more referrals to specialists (such as a hospital doctor or clinical psychologist) during routine follow-up, and 27% higher costs of treating recurrent breast cancer [27]. In contrast, a recent cost-consequence analysis of hospital versus telephone follow-up after treatment for endometrial cancer revealed that telephone follow-up was cost-neutral and was not associated with additional psychological morbidity, lower patient satisfaction, or reduced quality of life [28]. To the best of our knowledge, little is known about the HCU of patients with breast cancer who received ONN-led telephone follow-up through TMC. The purpose of this study was to characterize women who were consulted by ONNs through TMC, and to evaluate all-cause HCU of patients who received this service. In MHS, patients that received telephone-based consultation via TMC are those who were recommended for chemotherapy or immunotherapy with or without radiation and agree to be addressed by the ONN via TMC. Our single-payer setting enabled us to provide reliable and comprehensive measures of HCU data for a representative cohort of patients.

## Materials and methods

Part I of the study examined if there are differences in demographic or socio-economic characteristics between breast cancer patients who received telephone-based consultation of ONN via TMC and those who did not. Part II examined the annual HCU of patients who received telephone-based consultation of ONN via TMC at baseline (one year before first consultation) and one year following it.

### Study design and setting

A retrospective cohort study was conducted among MHS enrollees, the second largest HMO in Israel (with approximately 2.1 million enrollees [29] that is regulated by the National Health Insurance Law). The study was approved by the Institutional Ethics Committee (#0029-17-BBL).

### Study population

Part I: All female patients who were included in the MHS registry of breast cancer and were newly diagnosed in 2016 (n = 1035). Of those, 225 patients received telephone-based consultation of ONN via TMC (henceforth: TMC patients), and 810 patients had not (henceforth: non-TMC patients). In MHS, this service is designated to ≥25 years old women who receives chemotherapy or immunotherapy with or without radiation, that informed their physician that they are willing to be addressed by oncology nurse via TMC. This service is not applicable for: patients in terminal stage, patient who are only recommended to undergo lumpectomy, patients who are hospitalized, patients with psychiatric or other severe comorbidities, and patients who reached the treatment goals. Thus, while TMC patient are rather a homogeneous group, non-TMC patients are a more heterogenous one, that include patients in various disease severity (including severely ill patients) who may receive chemotherapy and/or immunotherapy and/or radiation therapy or those that undergo lumpectomy or receive adjuvant therapy. Nine subjects who received the service before diagnosis were excluded from the analyses.

Part II: All TMC patients whose first telephone-based consultation was between January and September 2016 (thus full data of 12 months of follow-up after first consultation was available in the database), and enrolled in MHS during the entire follow-up period (n = 110). Three subjects with outlier HCU data were excluded from the analyses.

### Healthcare utilization

MHS enrollees, like all Israeli citizens, are covered via generous and quite uniform universal health insurance that provides access to various healthcare services with no or relatively low copayments. HCU estimates analyzed in our study included: hospitalizations (by hospital ward, e.g., internal medicine or gynecology), primary care physician visits, diagnostic procedures and paramedical care of non-MHS health providers, medications, surgical procedures (by hospital ward, e.g., plastic surgeries or orthopedic surgeries), emergency room visits, and outpatient clinic visits (e.g., outpatient oncology care, imaging or oncologist visits). Total cost was calculated as the sum of all these estimates. Cost estimates were adjusted to 2017 prices and converted to US dollars (USD) using the August 2017 exchange rate of 3.6 Israeli shekels per 1 USD.

Annual HCU data were calculated for one year before and after first telephone-based consultation of ONN via TMC. The month of first telephone-based consultation of ONN via TMC was defined as the index date.

### Data analyses

Data were analyzed using STATA software (version 15.0, Stata Corp, College Station, TX, USA). The Mann-Whitney U test was used to determine between-group differences in age and socioeconomic status. A Chi-squared test was used to examine between-group differences in dichotomous variables (e.g., insurance coverage or comorbidities). The Wilcoxon signed-rank was used to determine within-group differences in HCU.

Since HCU estimates were not normally distributed, we specified a multivariable ordered logit model of the determinants of annual HCU costs by quarters of the distribution. Independent variables included in the model were: total annual number of telephone-based consultations (as a measure of the extent of exposure to TMC services), gap between diagnosis and first consultation, age, socio-economic status, eligibility for disability and income security benefits, and comorbidities. p-values of <0.05 determined statistical significance in all analyses.

## Results

### Part I: Characteristics of TMC and non-TMC patients

Table 1 presents a comparison of the demographic, clinical, and socio-economic characteristics between TMC patients (n = 225) and non-TMC patients (n = 810).

**Table 1:** Characteristics of TMC patients^a^ and non -TMC patients^a^.

	TMC patients^a^	Non-TMC patients^a^	p-value
**n**	225	810	
**Age**^**b**^	56.2 ± 11.9 (56, 27–82)	60.8 ± 12.9 (61, 27–95)	< 0.001^c^
**Heart disease %**	10.2	11.1	0.705^d^
**Diabetes %**	16.0	13.0	0.240^d^
**Hypertension %**	28.0	37.4	0.009^d^
**SES**^**b,e**^	6.3 ± 1.7 (6, 2–10)	6.5 ± 1.8 (7, 1–10)	0.090^c^
**Supplementary Insurance coverage %**			0.720^d^
**None (1)**	4.9	4.4	
**Magen Zahav (2)**	60.9	63.8	
**Maccabi Sheli (3)**	34.2	31.7	
**% who receives Disability benefits**	34.7	17.3	< 0.001^d^
**% who receives income security benefits**	9.8	15.9	0.021^d^

^a^ TMC patients- patients who received telephone-based consultation of ONN via TMC, non-TMC patients- patients who did not receive this service.

^b^ Values are mean ± SD (median, min-max)

^c^ Mann-Whitney U test

^d^ Chi-squared test

^e^ SES- socioeconomic status range between low (1) and high (10) and defined by the Israeli Central Bureau of Statistics according to place of residency.

TMC patients were significantly younger (56.2 vs. 60.8 years old, p<0.001), and had a lower prevalence of hypertension (28.0 vs. 37.4, p = 0.009). However, no significant difference was observed between groups in the prevalence of heart disease (10.2% vs. 11.1%, p = 0.705) or diabetes (16.0% vs. 13.0%, p = 0.240). Surprisingly, twice as high a proportion of TMC patients were receiving disability benefits compared to non-TMC patients (34.7% vs. 17.3%, p<0.001). The proportion of those who were receiving income security benefits was lower as well (9.8% vs. 15.9%, p = 0.021); however, no significant difference was observed between groups with regard to socio-economic status based on place of residency (levels 6.3 vs. 6.5, p = 0.090) and supplementary health insurance coverage (p = 0.720).

### Part II: HCU of TMC patients

One hundred seven TMC patients were analyzed in this part. Table 2 presents the detailed annualized HCU of TMC by type of service. The total average annual HCU of TMC patients before first consultation was $8857 and increased to $44130 in the first year following it (p<0.001). This increase stem predominantly from a significant increase in outpatient visits ($20380 vs. $3502, p<0.001) and medication costs ($19339 vs. $1758, p<0.001). Although more minor components of HCU, there were significant increases in primary care physician costs ($709 vs. $543, p<0.001) and hospitalization costs ($699 vs. $188, p = 0.016). No significant differences were observed in surgical procedure costs ($2603 vs. $2130, p = 0.622), diagnostic procedures and paramedical care of non-MHS health providers ($325 vs. $553, p = 0.653), and emergency room visits ($74 vs. $65, p = 0.148).

**Table 2:** Annualized healthcare utilization of TMC patients^a^ before and after first consultation.

	One year before first telephone-based consultation	One year after first telephone-based consultation	p-value^b^
**Total Healthcare** Costs	8857 **±** 6810 (7444, 4034–10683)	44130 ± 29145 (33034, 21538–72829)	<0.001
**Outpatient visits Costs**	3502 ± 2465 (2960, 1886–4280)	20380 ± 10096 (20354, 12858–26621)	<0.001
Number of visits	18.9 ± 21.7 (14, 9–21)	114.5 ± 134.9 (54, 34–136)	<0.001
**Medications** Costs	1758 **±** 3154 (762, 57–2148)	19339 ± 21509 (4064, 1285–38327)	<0.001
Number of Rx	29.7 **±** 24.2 (21, 12–38)	60.9 **±** 31.3 (57, 42–74)	<0.001
**Surgical procedures** Costs	2130 **±** 3697 (0, 0–1828)	2603 ± 4684 (0, 0–3786)	0.622
Number of procedures	0.7 **±** 1.0 (0, 0–1)	0.7 ± 1.0 (0, 0–1)	0.700
**Primary care Physician** Costs	543 ± 289 (508, 354–658)	709 ± 369 (628, 436–938)	<0.001
Number of visits	23.6 ± 12.3 (22, 16–28)	31.4 ± 15.3 (28, 20–42)	<0.001
**Acute Hospitalization** Costs	188 **±** 656 (0, 0–0)	699 ±1978 (0, 0–0)	0.016
Number of days	0.3 ± 1.1 (0, 0–0)	1.2 ± 4.0 (0, 0–0)	0.015
**Diagnostic procedures and paramedical care of non-MHS health providers** Costs	553 ± 1252 (0, 0–117)	325 ± 1510 (43, 0–190)	0.653
Number of procedures/visits	8.7 ± 8.2 (7, 3–11)	27.1 ± 13.9 (25, 17–35)	<0.001
**Emergency Room visits** Costs	65 ± 239 (0, 0–0)	74 ± 163 (0, 0–0)	0.148
Number of visits	0.3 ± 1.3 (0, 0–0)	0.3 ±0.7 (0, 0–0)	0.210
**Rehabilitation/geriatric Hospitalization** Costs	118 **±** 1220 (0, 0–0)	0 ± 0 (0, 0–0)	0.317
Number of days	0.2 ± 2.0 (0, 0–0)	0 ± 0 (0, 0–0)	0.317

Abbreviations: ONN- Oncology Nurse Navigator; TMC- Telemedical care

^a^ TMC patients- patients who received telephone-based consultation of ONN via TMC (n = 107)

^b^ Wilcoxon signed-rank test

Values are mean ±SD (median, 25 percentile-75 percentile). Cost estimates are in 2017 USD.

The costliest outpatient department was oncology, and it significantly increased among TMC patients following first consultation ($18413 vs. $679, p<0.001). The only significant increase in hospitalization costs was observed in the internal medicine ward ($310 vs. $71, p = 0.012). No significant increase was observed in hospitalizations in the plastic surgery ward ($134 vs. $0, p = 0.317), oncology ward ($83 vs. $8, p = 0.556), and the general surgical ward ($65 vs. $59, p0.991).

A multivariable ordered logit model of determinants of the total annual HCU costs is presented in Table 3. This analysis revealed that each additional telephone-based consultation of ONN via TMC increased HCU (OR = 1.07, 95% CI: 1.01–1.12, p = 0.013). Specifically, as presented in Table 4, each additional consultation decreased the likelihood to be in the lowest quarter of the HCU distribution by 1.1 percentage points (p = 0.015), and increased likelihood to be in the upper quarter of the HCU distribution by 1.1 percentage points (p = 0.016).

**Table 3:** Multivariable model of total annual health care costs of TMC patients^a^ (n = 107).

Model^b^	Ordered logit^c^		
	OR	95% CI	p-value
Annual number of telephone-based consultations (+1)	1.07	1.01–1.12	0.013
Gap between diagnosis and first consultation (in months)	0.78	0.63–0.95	0.015
Age (+1year)	0.96	0.92–1.00	0.060
Socioeconomic status (+1)^d^	1.23	0.95–1.58	0.111
Receiving income security benefits	1.32	0.18–9.61	0.783
Receiving disability benefits	0.80	0.34–1.89	0.610
Hypertension	0.80	0.31–2.06	0.637
R^2^/Pseudo R^2^	0.10		

^a^ TMC patients- patients who received telephone-based consultation of oncology nurse navigator via telemedical care.

^b^ The variable of interest is the annual number of telephone-based consultations. The model was adjusted for the gap between diagnosis and first consultation, age, socioeconomic status, receiving income security benefits, receiving disability benefits and having hypertension (in addition to breast cancer).

^c^ Categories for the dependent variables are: 1- The first lowest quarter; 2- The second quarter; 3- The third quarter; 4- The firth upper quarter.

^d^ Socioeconomic status range between low (1) and high (10) and defined by the Israeli Central Bureau of Statistics according to place of residency.

**Table 4:** The predicted change in the probability of being in each of HCU quarters following each additional telephone-based consultation.

	First lowest quarter	Second quarter	Third quarter	Fourth upper quarter
Additional telephone-based consultation	-0.011	-0.005	0.005	0.011
p-value	0.015	0.062	0.047	0.016

ONN- oncology nurse navigator; TMC- telemedical care; HCU- Healthcare utilization.

Finally, the average gap between diagnosis and first consultation was 2.3 month (median = 2.4 months, and maximum = 6.1 months). Since the first year following diagnosis is considered the costliest, and since the HCU costs were estimated for the first year following first consultation (rather than the first year following diagnosis) it was important to include in the multivariable model, the gap between diagnosis and first consultation. Indeed, as expected and presented in Table 3, we revealed that a longer gap decreased the likelihood of elevated HCU (OR = 0.78, 95% CI: 0.63–0.95, p = 0.015).

## Discussion

This study reveals that only 22% of our cohort received telephone-based consultation of ONN via TMC, and significant demographic and socio-economic differences were found between these patients and those who did not receive this service. Specifically, TMC patients were younger and had a lower prevalence of hypertension than the non-TMC patients. A higher proportion of these patients received disability benefits, and a lower proportion received income security benefits. In addition, the analysis of HCU of those who received telephone-based consultations of ONN via TMC revealed that HCU significantly increased among this group, mainly due to increased utilization of outpatient visits and medications. A multivariable model revealed that a more intense use of telephone-based consultation of ONN via TMC was associated with higher HCU. These results will be discussed in light of the currently available literature.

Part I of the study was targeted at characterizing breast cancer patients who received telephone-based consultations of ONN via TMC. We found that approximately one-fifth of our cohort received this service; but unfortunately, data of the response rate of the TMC of MHS were not available. In fact, other studies have reported suboptimal response rates. For example, the response rate reported in an 11-hospital health system in southeast Texas that routinely performed calls after patient discharge was 47%, with rates for individual nursing units ranging from 33% to 59% [30]. The percent of individuals who were successfully contacted for follow-up after burn injury (via phone calls, mailings, and clinic visits) was 64% at 6 months and 54% at 12 months [31]. Another study that was targeted at recruiting participants for genetic counseling and testing for inherited breast cancer revealed that, in 42% of eligible patients who had invasive breast cancer ≤50 years old identified by the Florida State Cancer Registry, telephone contact with a genetic counselor was established [32]. A study that evaluated the feasibility of a call-in center to deliver colorectal cancer screening via telephone outreach revealed that 32.9% of the study cohort contacted the call-in center following a mailed invitation [33]. Following these less than ideal response rates and due to the potential benefits associated with telephone-based follow-up, it seems that monitoring the response rate of this service is crucial to properly evaluate its effectiveness.

We found that breast cancer patients who had received telephone-based consultations of ONN via TMC were significantly younger compared to those who had not received this service. Different results were found by D’Amore et al. [30], where patients discharged with various diseases who were successfully contacted by telephone follow-up were significantly older. Similarly, it was found that individuals who were younger were lost to follow-up after burn injury [31]. On the other hand, no differences were found in clinical and demographic characteristics between breast cancer patients who were contacted by a genetic counselor and those who were not [32].

Unexpectedly, we found that a lower proportion of TMC patients were receiving income security benefits. Similarly, it was found that individuals who were not employed at the time of their burn injury were lost to follow-up [31]. However, it should be noted that we found no significant difference in other proxies of socio-economic status based on place of residency or supplementary health insurance coverage. These mixed results and the growing use of different follow-up technologies highlight the need for further research to examine factors associated with loss to follow-up.

Part II of our study analyzed HCU of patients who received telephone-based consultation of ONN via TMC. We found that 90% of the total HCU costs following first consultation were attributed to outpatient care (mostly visits to the oncology outpatient clinic) and medications (46% and 44% of total costs, respectively). Our administrative database did not include pharmacological details, yet we assume that these costs mainly refer to chemotherapy or immunotherapy. In a study of total HCU during all the disease course across European countries, it was found that relatively similar to our estimation, medication costs made up 46% of total costs; however, the proportion of outpatient visits was 9%, significantly lower [4]. Research that analyzed the HCU of metastatic breast cancer patients receiving chemotherapy revealed that 29% of HCU costs were attributed to outpatient visit costs and 51% were attributed to medication costs [7]. Although this result may seem different, one should note that total outpatient and medication costs composed a similar proportion of total costs in both studies (90% in our study and 80% in the Vera-Llonch et al. study); thus, it is assumed that reimbursement schemes may attribute the same service (e.g., chemotherapy) to different cost components (e.g., outpatient visits instead of medication). In addition, our results are similar to those estimated by Allaire et al. for the annual HCU costs during all of the disease course, where outpatient care that included medications (such as hormonal therapies, and neoadjuvant and adjuvant medications), surgical procedures (such as lumpectomy), radiation therapies, and physician visits were 92% of the total HCU costs for breast cancer [3], similar to our results, where outpatient visits (46%), medication (44%), and surgical procedures (6%) composed 96% of the total HCU. However, it must be noted that, while our results were derived from a mostly publicly-financed HMO, those of Vera-Llonch et al. [7] and Allaire et al. [3] were derived from privately-financed healthcare.

Total annual hospitalization costs were found to be negligible in our cohort, similar to the results of Allaire et al. [3]. However, the studies of Luengo-Fernandez et al. from the European countries [4] and Barron et al. from the US [2] revealed that 39% and 46% of costs, respectively, were attributed to inpatient care. This difference may stem from the fact that, different from our analysis that focused on the first year following first consultation (which was relatively close to the diagnosis month), the former two studies provided an analysis of HCU of patients at different points in the disease course. Thus, that analysis may include the “terminal phase” of the disease which was estimated as costly (in addition to the initial phase immediately following diagnosis), and includes a high hospitalization rate [11]. In addition, this difference may be attributed to differences in cancer types, disease severity, and differences in practice patterns [2, 4]. It should be noted that, in Israel, patients with breast cancer have mainly the invasive disease [34]; thus, it may be assumed that a long-term analysis of HCU during the whole disease course might result in a significant proportion of hospitalizations. Our results portray different patterns of HCU. Namely, in the first year following first consultation of ONN, ambulatory care is much more significant than inpatient care.

The direct HCU cost represents only one part of the total financial burden of breast cancer. From a societal perspective, this disease has a financial burden also attributed to indirect costs such as earnings lost due to illness or death, cost of support by families and friends, and travel costs associated with care. Research among European countries revealed that these indirect costs made up 65% of the total economic burden of the disease [4], while another study from Flanders estimated this portion at 89% [9]. An additional burden of breast cancer stems from out-of-pocket expenditures [5]; hence, healthcare systems worldwide should focus efforts on analyzing this excess burden, find ways to reduce HCU costs, and enable generous universal coverage [35].

Our study revealed that a more intense use of TMC was associated with elevated HCU. This trend is similar to that observed by Beaver et al. [27], who found that compared to hospital-based consultations, nurse-led telephone follow-up resulted in more referrals to specialists (such as hospital, doctor, or clinical psychologist) during routine follow-up and 27% higher costs in treating recurrent breast cancer [27]. However, results from the study of Porath et al., in the same setting as ours (TMC in MHS), revealed a significant reduction in hospitalizations for frail chronic elderly patients who had received this service [25]. This difference may stem from the different nature of HCU of chronically ill patients and those with breast cancer, and the corresponding role of TMC in these two populations. Inpatient care is the largest component of total HCU costs among patients with chronic conditions [36, 37]. On the other hand, our study revealed that outpatient care is the largest component of total HCU costs of patients with breast cancer 12 months following first ONN consultation. In addition, a systematic review that examined the association between chronic disease-related hospitalizations and primary healthcare resourcing provided the impression that access to quality primary care may be associated with a reduced rate of avoidable hospitalizations [38]. Thus, while TMC may provide a qualitative and efficient service that may reduce avoidable hospitalizations of chronically ill patients in general, when provided to breast cancer patients during the first year following diagnosis, rather than a substitute for care, TMC actually facilitates more usage of it (such as medication and outpatient visits); thus, this service actually increases HCU. Even so, in this context, it may reduce the burden on scarce hospital resources [27].

A systematic review that focused on technologies used for cancer follow-up revealed that this technology did not compromise patient satisfaction or safety, as measured by symptoms, health-related quality of life, or psychological distress [24]. Previous studies have described how ONN services support cancer patients [12]. It was shown that this service provides timely care to alleviate problems and improve disease management [13], provides support and information in distant places of residency [14], and improves patient satisfaction and mental wellbeing [15, 16]. However, the influence of ONN and TMC in terms of decreased HCU was inconsistent [22]. There was evidence that exposure to an ONN decreased hospitalization in head and neck cancer [20], yet increased [27] or did not change costs [21] in breast cancer, and did not change costs in endometrial cancer [28]. The difference between our study and others may stem from several reasons. First, we analyzed patients in their first year after diagnosis, which is known to be the costliest, and at this point in the disease course, patients might be more proactive and engaged in disease management, and thus more cooperative with the ONN who facilitates their care. Second, our cohort was relatively young; thus, they might have higher mobility and health literacy that are crucial to the actual consumption of healthcare services. Finally, because all patients in our cohort were covered by universal health insurance, following ONN services that guided the patient to optimal use of various services, almost no additional barrier was present.

This study has several limitations. First, our analysis relied on financial data that lack sufficient clinical information to confirm the disease stage and severity at diagnosis and before the telephone-based follow-up via TMC. Thus, we could not stratify the results into disease severity and treatment subgroups, and we acknowledge that these clinical characteristics might contribute to variability in the HCU estimates. Second, HCU in our study includes direct HMO cost and ignore out of pocket costs for medication or other healthcare services. Third, cost estimates of HCU may not be generalizable to other healthcare systems, as practice patterns and tariffs may differ. This limitation, however, does not weaken our analysis since our objective was to examine the determinants of elevated HCU following first consultation, rather than to refer to absolute values. Finally, although our cohort was quite large and represented all patients diagnosed with breast cancer in the second largest HMO in Israel in 2016, in Part II of the study, the actual number of participants was relatively small, and thus may not include a representative sample.

Notwithstanding these limitations, we believe that our study contributes to the extant knowledge in two main ways. First, it provides a comprehensive description of all-cause healthcare costs of breast cancer patients who receive telephone-based consultation of ONN via TMC in Israel, thus, substantiating existing evidence with regard to the economic burden of breast cancer worldwide. Second, it provides a unique analysis of the influence of exposure to this service in terms of the likelihood to have elevated HCU. Further research on long-term HCU and health outcomes of patients with breast cancer who receives telephone-based consultation of ONN via TMC will enable more in-depth examination of the effectiveness of this service for this particular disease.
